# Burden of dilated perivascular spaces in patients with moyamoya disease and moyamoya syndrome is related to middle cerebral artery stenosis

**DOI:** 10.3389/fneur.2023.1192646

**Published:** 2023-06-05

**Authors:** Guangsong Han, Xiaoyuan Fan, Yuehui Hong, Lixin Zhou, Yicheng Zhu, Feng Feng, Ming Yao, Jun Ni

**Affiliations:** ^1^Department of Neurology, Peking Union Medical College Hospital, Peking Union Medical College and Chinese Academy of Medical Sciences, Beijing, China; ^2^Department of Radiology, Peking Union Medical College Hospital, Peking Union Medical College and Chinese Academy of Medical Sciences, Beijing, China

**Keywords:** moyamoya disease, moyamoya syndrome, perivascular space, large artery stenosis, cerebral atrophy

## Abstract

**Background and objective:**

The correlation between intracranial large artery disease and cerebral small vessel disease (CSVD) has become a noteworthy issue. Dilated perivascular spaces (dPVS) are an important marker of CSVD, of which cerebral atrophy has been regarded as one of the pathological mechanisms. DPVS has been found to be associated with vascular stenosis in patients with moyamoya disease (MMD), but the underlying mechanism remains unclear. The purpose of our study was to explore the correlation between the middle cerebral artery (MCA) stenosis and dPVS in the centrum semiovale (CSO-dPVS) in patients with MMD/moyamoya syndrome (MMS) and to determine whether brain atrophy plays a mediating role in this relationship.

**Methods:**

A total of 177 patients were enrolled in a single-center MMD/MMS cohort. Images of their 354 cerebral hemispheres were divided into three groups according to dPVS burden: mild (dPVS 0–10), moderate (dPVS 11–20), and severe (dPVS > 20). The correlations among cerebral hemisphere volume, MCA stenosis, and CSO-dPVS were analyzed, adjusting for the confounding factors of age, gender, and hypertension.

**Results:**

After adjustment for age, gender, and hypertension, the degree of MCA stenosis was independently and positively associated with ipsilateral CSO-dPVS burden (standardized coefficient: β = 0.247, *P* < 0.001). A stratified analysis found that the subgroup with a severe CSO-dPVS burden exhibited a significantly higher risk of severe stenosis of the MCA [*p* < 0.001, OR = 6.258, 95% CI (2.347, 16.685)]. No significant correlation between CSO-dPVS and ipsilateral hemisphere volume was found (*p* = 0.055).

**Conclusion:**

In our MMD/MMS cohort, there was a clear correlation between MCA stenosis and CSO-dPVS burden, which may be a direct effect of large vessel stenosis, without a mediating role of brain atrophy.

## 1. Introduction

Since cerebral small vessel disease (CSVD) shares common vascular risk factors with large artery disease, the correlation between intracranial large artery disease and the markers of CSVD has recently been explored. Previous studies have shown that intracranial atherosclerotic stenosis is independently associated with the burden of progressive severe white matter hyperintensities (WMHs), and this association appears to be stronger than the association with extracranial atherosclerotic stenosis in the Asian stroke population (1). Middle cerebral artery or basilar artery atherosclerotic stenosis might lead to chronic cerebral hypoperfusion, which could give rise to cerebral microbleeds (CMBs) (2). Intracranial atherosclerotic stenosis generally affecting the proximal perforating arteries is among the major pathologies underlying lacunes (3). Our previous findings indicated that ischemic imaging markers of CSVD, including lacunes, WMHs, and brain atrophy, were associated with intracranial arteriosclerosis in a large population-based cohort (4). Additionally, carotid atherosclerosis and dilation are associated with lacunes and larger WMHs (5).

Perivascular spaces (PVS), which are also known as Virchow–Robin spaces, are described as the spaces surrounding the cerebral perforating arteries and veins coursing from the subarachnoid space into the brain parenchyma (6). Dilated perivascular spaces (dPVS) are commonly detected as dot-like or linear hyperintensities (generally with a maximum diameter of < 3 mm) on T2-weighted MRI; they are most commonly found in the basal ganglia, centrum semiovale, and midbrain (6, 7). dPVS have previously been found to be associated with aging and hypertension (6) and are now recognized as an important imaging marker of cerebral small vessel disease (CSVD) due to their strong and independent relationship with lacunar stroke and WMHs (6, 8). Limited researches clarifying the relationship between dPVS and intracranial arterial stenosis are available with inconsistent observations. dPVS was not identified as being related to intracranial atherosclerotic stenosis in our previous report based on a large population-based cohort (4). In contrast, a biological link between atherosclerotic stenosis and increased dPVS has previously been reported in another study (9).

Moyamoya disease (MMD) is a rare cerebrovascular disease characterized by progressive stenosis of the internal carotid arteries and their proximal branches, accompanied by an abnormal vascular network at the base of the brain. Patients with the characteristic cerebrovascular features of MMD in the context of a definite underlying risk factor or disease are categorized as having moyamoya syndrome (MMS) (10). MMD/MMS is considered to be a typical model of intracranial artery stenosis, while several imaging markers of CSVD, including lacunes, WHMs, cerebral microbleeds, and dPVS, are observed in MMD patients (11). MMD/MMS is thus of great value in the evaluation of the correlation between large artery stenosis and small vessel disease. dPVS in MMD patients have previously been found to be associated with female gender, hypertension, vascular stenosis measured by magnetic resonance angiography (MRA), decreased regional blood perfusion, and cerebral atrophy (7, 11), but the specific mechanisms underlying these correlations remain ambiguous. The traction of atrophic brain tissue on perivascular tissue is considered to account for the correlation between brain atrophy and basal ganglia dPVS observed in lacunar infarction patients (12). Additionally, Mestre et al. found that the obstruction of the cerebral lymphatic fluid system caused by cerebral hypoperfusion is among the most important mechanisms of PVS (7, 8). However, the exact pathogenic mechanism responsible for the relationship between intracranial vascular stenosis and dPVS in MMD/MMS patients remains to be clarified.

The aim of the present study was to explore the correlation between dilated perivascular spaces in the centrum semiovale (CSO-dPVS) and the degree of MCA stenosis in a large cohort of MMD/MMS patients, and further to determine whether brain atrophy plays a mediating role in this possible correlation.

## 2. Materials and methods

### 2.1. Patients

Data were obtained from a single-center prospective cohort. A total of 177 patients with MMD or MMS were consecutively enrolled between September 2017 and December 2022 from the neurology department of Peking Union Medical College Hospital. In all cases, the presence of moyamoya vasculopathy was confirmed according to the guidelines published in 2012 (13) (MRA shows stenosis or occlusion of the terminal portion of the intracranial internal carotid artery or proximal portions of the anterior and/or the middle cerebral artery and abnormal vascular networks in the basal ganglia). All patients presenting with this typical vasculopathy, with or without associated risk factors, were enrolled. Underlying associated risk factors included atherosclerosis, autoimmune disease, hyperthyroidism, meningitis, brain tumors, head injury, and head irradiation. Clinical and demographic data were collected, including age, gender, clinical events associated with MMD/MMS, and medical history (hypertension, hyperlipemia, thyroid disease, smoking, and alcohol consumption).

### 2.2. MRI and MRA examinations

MRI scans were performed with the use of a single 3.0-T system. The 354 brain hemispheres of 177 patients with MMD/MMS were examined. The following MRI sequences were used for assessment: T1-weighted imaging (T1WI); T2-weighted imaging (T2WI); fluid-attenuated inversion recovery (FLAIR); diffusion-weighted imaging (DWI); time-of-flight MR angiography (TOF MRA); and high-resolution vessel wall imaging (HR VWI). The parameters for the 3.0T scanner were as follows. T1WI sequence: repetition time (TR), 1,775 ms; echo time (TE), 21.6 ms; inversion time (TI), 720 ms; flip angle (FA), 111°; slice thickness, 4 mm; number of slices, 40; acquisition matrix, 320 × 160; field of view (FOV), 240 × 168; and b_max_, NA. T2WI sequence: TR, 5,258 ms; TE, 84 ms; TI, NA; FA, 142°; slice thickness, 4 mm; number of slices, 36; acquisition matrix, 320 × 320; FOV, 220 × 220; and b_max_, NA. FLAIR sequence: TR, 12,000 ms; TE, 122 ms; TI, 2,712 ms; FA, 160°; slice thickness, 4 mm; number of slices, 36; acquisition matrix, 288 × 192; FOV, 220 × 176; and b_max_, NA. DWI sequence: TR, 3,600 ms; TE, 64 ms; TI, NA; FA, 90°; slice thickness, 4 mm; number of slices, 36; acquisition matrix, 128 × 128; FOV, 220 × 220; and b_max_, 1,000 s/mm^2^. 3D TOF MRA sequence: TR, 16 ms; TE, 2.1 ms; TI, NA; FA, 20°; slice thickness, 1.2 mm; number of slices, 288; acquisition matrix, 256 × 256; FOV, 220 × 220; and b_max_, NA. 3DT1 sequence: TR, 6.7 ms; TE, 2.9 ms; TI, 400 ms; FA, 12°; slice thickness, 1.0 mm; number of slices, 170; acquisition matrix, 256 × 256; FOV, 256 × 230; and b_max_, NA. HR VWI sequence: TR, 800 ms; TE, 15.8 ms; TI, NA; slice thickness, 0.8 mm; number of slices, 248; acquisition matrix, 320 × 256; FOV, 204 × 184; and b_max_, NA.

#### 2.2.1. Assessment of dPVS

**Figure 1:**
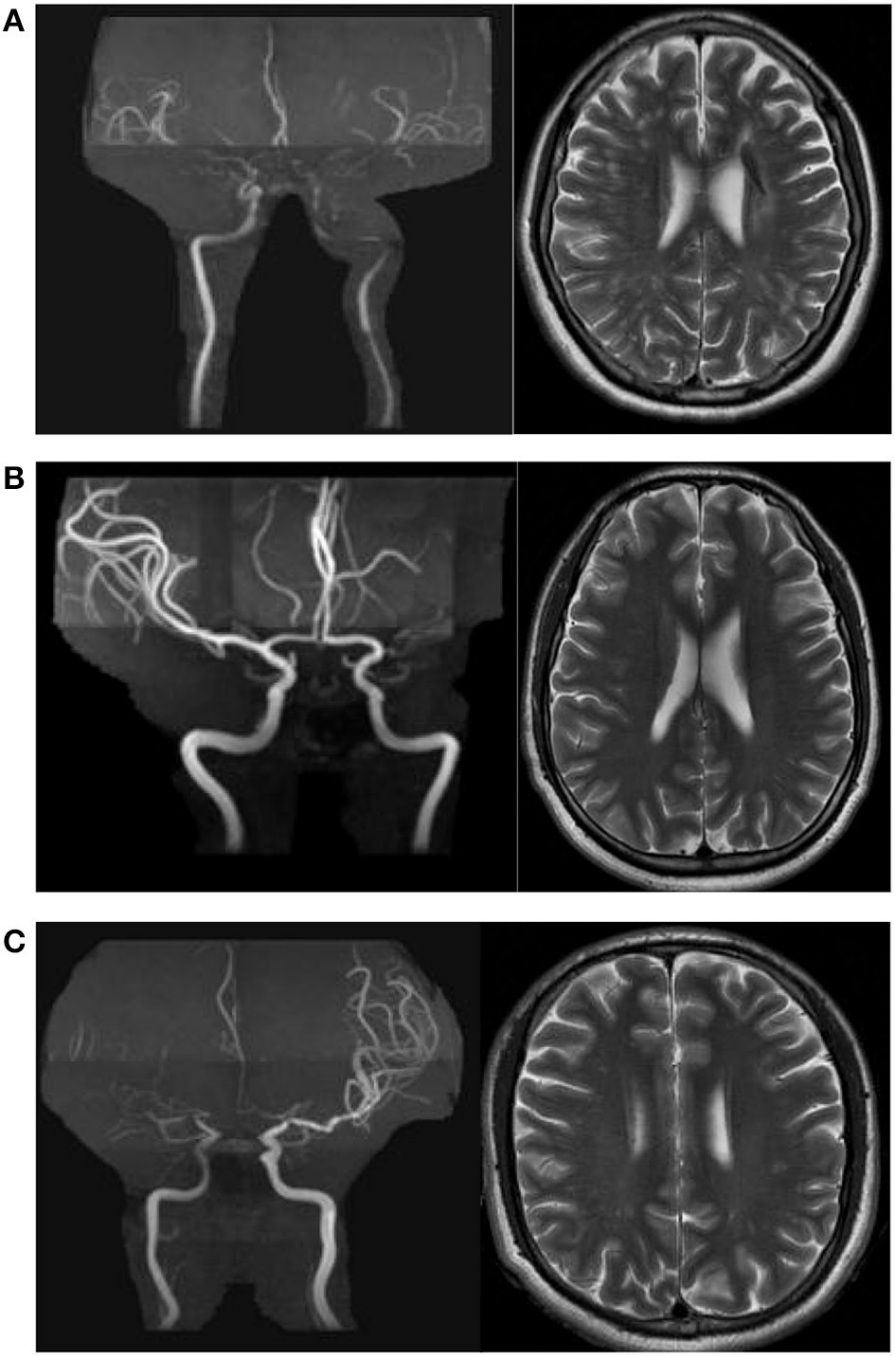
Examples of centrum semiovale dilated perivascular spaces (CSO-dPVS) of different severity. **(A)** 54/F, ischemic stroke; MRA shows bilateral MCA occlusion. The CSO-dPVS burden is severe bilaterally (dPVS > 20). **(B)** 35/F, recurrent episodes of dizziness; MRA shows left MCA occlusion, and no stenosis is observed in the right MCA. The left CSO-dPVS burden is severe (dPVS > 20) and the right CSO-dPVS burden is mild (dPVS 0–10). **(C)** 33/M, recurrent episodes of dizziness; MRA shows right MCA occlusion, and moderate stenosis is observed in the left MCA. The right CSO-dPVS burden is severe (dPVS > 20) and the left CSO-dPVS burden is moderate (dPVS 11–20).

A dilated PVS was defined as a lesion with CSF-like signal-intensity (hypointense on T1 and hyperintense on T2) that was round, ovoid, or linear, < 3 mm in its maximum diameter, with smooth delineated contours, and located in an area supplied by perforating arteries (14). The dPVS burden was evaluated at the level of the centrum semiovale on T2-weighted images. Basal ganglia dPVS (BG-dPVS) was not evaluated in the present study because the accuracy of evaluation of BG-dPVS on axial T2WI is limited due to the presence of characteristic flow voids and lacunes in the basal ganglia in MMD, as mentioned previously (7, 11). All 354 hemispheres were categorized into one of three subgroups by severity of CSO-dPVS burden (15) (Figures 1A–C): mild (CSO-dPVS 0–10), moderate (CSO-dPVS 11–20), or severe (CSO-dPVS > 20). All images were analyzed by the same experienced reader (GSH), who was blinded to the clinical data. When the reader was uncertain, the lesions were reviewed by another reader with more experience in MR imaging studies (MY), and a decision was made by consensus. Intra-rater agreement for the rating of dPVS was assessed on a random sample of 20 subjects after a 1-month interval; this analysis showed a good level of reliability, with a kappa value of 0.82.

#### 2.2.2. Assessment of stenosis of MCA

The 354 MCAs were divided into three groups according to the severity of MCA stenosis on MRA and HR VWI: without stenosis; mild or moderate stenosis (0–69%); and severe stenosis or occlusion (70–100%). All MRA images were analyzed by the same experienced reader (YHH), who was blinded to the clinical data.

#### 2.2.3. Assessment of brain atrophy

The Dicom Sort software package was used to convert the format of the 3DT1 sequences from DICOM to NIFTI. Computational Anatomy Toolbox (CAT) 12 was used to segment the left and right hemispheres and to construct a Montreal Neurological Institute (MNI) space hemisphere mask. Finally, the masks of the left and right hemispheres were applied to the segmentation results to obtain measures of the bilateral gray matter and white matter volumes. All volumes for the 354 hemispheres were analyzed by the same experienced reader (GSH), who was blinded to the clinical data.

### 2.3. Statistical analysis

Continuous variables are summarized in the form of means with the corresponding standard deviation (SD); categorical variables are summarized as percentages. Each data was analyzed separately over 354 independent hemispheres from 177 patients with MMD/MMS. First, we explored the relationships between CSO-dPVS and potential risk factors (including age; gender; history of hypertension, diabetes, and hyperlipidemia; degree of MCA stenosis, and ipsilateral hemisphere volume) using univariate analysis. Nonparametric Spearman correlation tests were used for continuous variables; *t*-tests were used to compare the mean CSO-dPVS for categorical variables. Second, a multivariate linear regression model was used to investigate the correlation between CSO-dPVS burden and degree of MCA stenosis, with CSO-dPVS burden as the dependent variable.

Candidate covariates included age, gender, and hypertension. To further explore the relationship between dPVS burden and degree of MCA stenosis, a logistic regression model was constructed for dPVS burden category, using the mild group as the reference category. All of the abovementioned candidate covariates were included. Finally, a multivariate linear regression model was constructed to investigate the correlation between CSO-dPVS and cerebral volume, with CSO-dPVS as the dependent variable and including the abovementioned candidate covariates. The degree of MCA stenosis was additionally included in the model. Statistical analyses were performed using SPSS version 24.0 for Windows (SPSS Inc.). All *p*-values were two-tailed, and the criterion for statistical significance was *p* < 0.05.

## 3. Results

### 3.1. Demographic characteristics and potential risk factors related to the severity of CSO-dPVS burden

The characteristics of the patients are presented in Table 1. The mean age was 42.33 ± 12.11 years. Of these 177 individuals, 95 had ischemic stroke, 12 had hemorrhagic attacks, and 70 were asymptomatic or reported only headache or other non-specific symptoms. Data on all patients' risk factors for cerebrovascular disease were collected.

**Table 1:** Main characteristics of the cohort (*N* = 177)^*^.

**Demographics**	
Age, mean (SD)	42.33 (12.11)
Male gender	57 (32.2%)
Hypertension	64 (36.2%)
Hyperlipemia	13 (7.3%)
Diabetes	23 (13.0%)
Smoking	23 (20.3%)
Alcohol consumption	26 (15.3%)
Hyperthyroidism	11 (6.2%)
History of cranial and cervical radiation therapy	1 (0.6%)

^*^All data are presented in the form number (percentage) unless otherwise indicated.

In the univariate analysis, CSO-dPVS burden was positively related to age (Spearman ρ = 0.112, *p* = 0.035). Additionally, patients with hypertension were found to have a higher CSO-dPVS burden (13.96 ± 7.85 vs. 11.27 ± 8.07, *p* < 0.001). CSO-dPVS burden was also correlated with degree of MCA stenosis (*p* < 0.001) but was not significantly related to ipsilateral hemisphere volume (*p* = 0.086). Gender, current smoking status, alcohol consumption, and history of hyperlipidemia and diabetes were not correlated with CSO-dPVS burden (all *p*-values > 0.05).

### 3.2. Multivariate analysis of the correlation between CSO-dPVS burden and degree of MCA stenosis

After adjustment for age, gender, and hypertension, degree of MCA stenosis was independently and positively associated with number of ipsilateral CSO-dPVS (standardized coefficient: β = 0.247, *p* < 0.001, as shown in Table 2). Hypertension was also significantly associated with CSO-dPVS [95% CI (0.362, 3.865), β = 0.140, *p* = 0.009, as shown in Table 2].

**Table 2:** Multivariate linear regression examining the correlation between CSO-dPVS burden and MCA stenosis^*^.

	**Estimate 95% CI**	**Standardized coefficient (β)**	***P*-value**
Age	(−0.030, 0.109)	0.043	0.418
Male gender	(−1.681, 1.814)	0.027	0.594
Degree of MCA stenosis	(1.432, 3.299)	0.247	**< 0.001**
Hypertension	(0.362, 3.865)	0.140	**0.009**

^*^A multivariate linear regression analysis was conducted to assess the association between CSO-dPVS burden and MCA stenosis. CSO-dPVS burden was taken as the dependent variable. Adjustments: age, gender, and hypertension.

MCA, middle cerebral artery; CSO-dPVS, centrum semiovale dilated perivascular spaces.

The meaning of the bold values is *P* < 0.05.

To further explore the relationship between dPVS burden and degree of MCA stenosis, hemispheres were categorized into three subgroups by severity of dPVS burden, based on the number of CSO-dPVS. The potential risk factors and radiological characteristics are summarized for each of the three subgroups in Table 3. Compared with the mild CSO-dPVS burden subgroup, the severe burden subgroup exhibited a significantly higher risk of severe stenosis of the MCA [*p* < 0.001, OR = 6.258, 95% CI (2.347, 16.685), as shown in Table 4]. A similar association was also found for the moderate CSO-dPVS burden subgroup [*p* = 0.005, OR = 2.187, 95% CI (1.262, 3.791)], but with a relatively lower hazard (shown in Table 4).

**Table 3:** Raw distribution of potential risk factors and radiological characteristics by severity of CSO-dPVS burden^*^.

	**Subgroups by CSO-dPVS burden**		
	**Mild burden**	**Moderate burden**	**Severe burden**
	***N*** = **175**	***N*** = **122**	***N*** = **57**
**Demographics**			
Age, mean (SD)	41.35 (0.976)	42.61 (0.993)	43.08 (11.97)
Male gender	52 (29.7%)	42 (34.4%)	20 (35.1%)
Hypertension	48 (27.4%)	53 (43.4%)	27 (47.4%)
Hyperlipemia	18 (10.3%)	8 (6.6%)	0 (0.0%)
Diabetes	15 (8.6%)	19 (15.6%)	12 (21.1%)
Smoking	34 (19.4%)	28 (23.0%)	10 (17.5%)
Alcohol consumption	25 (14.3%)	17 (13.9%)	12 (21.1%)
Hyperthyroidism	9 (5.1%)	10 (8.2%)	3 (5.2%)
**Radiological characteristics**			
Ipsilateral hemisphere volume, mean (SD)	15,704,650.57 (82,944.22)	15,688,475.69 (97,093.884)	15,647,065.66 (1,173,475.03)
**MCA stenosis**			
Without stenosis	61 (34.9%)	26 (21.3%)	5 (8.8%)
Mild or moderate stenosis	21 (12.1%)	7 (5.7%)	2 (3.5%)
Severe stenosis	93 (53.1%)	89 (73%)	50 (87.7%)

^*^All data are presented in the form of percentages or standard deviations (SD) unless otherwise indicated.

MCA, middle cerebral artery; CSO-dPVS, centrum semiovale dilated perivascular spaces.

**Table 4:** Subgroup analysis: logistic regression examining the correlation between CSO-dPVS burden and MCA stenosis^*^.

		**Estimate 95% CI**	**OR**	***P*-value**
Moderate burden	Age	(0.980, 1.021)	1.001	0.948
	Female gender	(0.529, 1.479)	0.885	0.641
	Hypertension	(0.311, 0.871)	0.520	**0.013**
	MCA: severe stenosis or occlusion	(1.262, 3.791)	2.187	**0.005**
	MCA: mild or moderate stenosis	(0.303, 2.151)	0.807	0.886
	MCA: without stenosis	Ref		
Severe burden	Age	(0.987, 1.041)	1.014	0.317
	Female gender	(0.437, 1.684)	0.858	0.656
	Hypertension	(0.253, 0.951)	0.491	**0.035**
	MCA: severe stenosis or occlusion	(2.347, 16.685)	6.258	**< 0.001**
	MCA: mild or moderate stenosis	(0.216, 6.748)	1.206	0.831
	MCA: without stenosis	Ref		

^*^In the polytomous logistic regression model, severity of CSO-dPVS burden was taken as the dependent variable, with the mild subgroup (CSO-dPVS: 0–10) as the reference category.

^*^Model: adjusted for age, gender, and hypertension.

^*^MCA, middle cerebral artery; CSO-dPVS, centrum semiovale dilated perivascular spaces.

The meaning of the bold values is *P* < 0.05.

### 3.3. Correlation between CSO-dPVS and ipsilateral hemisphere volume

After adjustment for age, gender, and hypertension, no significant correlation between CSO-dPVS burden and ipsilateral hemisphere volume was found (*p* = 0.055, as shown in Table 5). This association remained non-significant even with further adjustment for severity of MCA stenosis (data not shown).

**Table 5:** Multivariate linear analysis examining the correlation between CSO-dPVS burden and hemisphere volume^*^.

	**Standardized coefficient (β)**	***P*-value**
Age	0.096	0.082
Gender	−0.034	0.557
Hypertension	0.134	**0.015**
Hemisphere volume	−0.113	0.055

^*^A multivariate linear regression analysis was conducted to assess the association between CSO-dPVS burden and hemisphere volume. CSO-dPVS burden was taken as the dependent variable.

^*^Model: adjusted for age, gender, and hypertension.

CSO-dPVS, centrum semiovale dilated perivascular spaces.

The meaning of the bold values is *P* < 0.05.

## 4. Discussion

To our knowledge, this is the largest study analyzing the association between dPVS and arterial stenosis in MMD or MMS. A positive association between degree of MCA stenosis and CSO-dPVS burden was observed in the present cohort, in line with results previously observed in a limited number of patients (7). However, hemisphere volume was not found to be independently related to ipsilateral CSO-dPVS burden, which is inconsistent with previous findings that brain atrophy is involved in the pathogenesis of dPVS (12). These findings strongly suggest that the correlation between MCA stenosis in MMD/MMS and CSO-dPVS burden is not mediated by brain atrophy. Interestingly, the significant association between age and dPVS burden observed in the univariate analysis disappeared in our multivariate analysis, in contrast with a previous finding in which the degree of dPVS burden was positively and independently associated with age (16). At a cross-sectional level, the association between age and dPVS burden might be concealed by the effect of MCA stenosis on the development of dPVS in this relatively young cohort. This further highlights the strong association between MCA stenosis in MMD/MMS and CSO-dPVS burden.

Previous studies have focused on CSVD markers in ischemic stroke patients with intracranial and extracranial large artery atherosclerotic stenosis (4, 5). As a group of diseases with isolated intracranial large artery involvement, MMD/MMS is closer to an ideal model for investigation of the relationship between intracranial large artery stenosis and burden of CSVD markers. In recent years, with an increasing number of patients with MMD undergoing MRI examination and the advent of more in-depth research on MMD imaging, many CSVD imaging markers, such as lacunes, WMHs, CMBs, and dPVS, have been found in MMD patients. Certain mechanisms underlying CSVD markers in moyamoya disease have been preliminarily defined in previous studies: axonal destruction and glial proliferation caused by ischemic injury due to chronic hypoperfusion are the main mechanisms of WMHs in MMD/MMS patients; secondary to cerebral ischemia and decreased cerebral blood flow (CBF), increased hemodynamic burden is associated with multiple cerebral microbleeds (17, 18). Although an association between dPVS and decreased regional blood perfusion caused by intracranial artery stenosis has also been found in MMD (7), the main mechanism remains unclear. The relationship between BG-dPVS and MCA atherosclerosis is attributed to hypoperfusion caused by severe occlusive MCA, which could trigger hypoxia and impair the interstitial fluid drainage system, thereby ultimately facilitating the formation of BG-EPVS (19, 20). Moreover, there is also evidence to support the possibility that extravascular dilatation secondary to shrinkage of the cerebral tissue may be associated with the development of dPVS (12). Therefore, the mechanisms mentioned above may both be potential causes of dPVS in patients with MMD/MMS.

A strong correlation between CSO-dPVS burden and large artery stenosis in MMD/MMS was found in the present study, while no significant association between hemisphere volume and CSO-dPVS burden was detected. These findings indicated that a possible explanation for the association between CSO-dPVS and vascular stenosis in MMD/MMS patients might be diminished cerebral hypoperfusion, rather than brain atrophy playing a mediating role. Based on previous research, we speculate that the possible mechanism underpinning increased dPVS burden in patients with MMD/MMS is as follows: the structural basis of dPVS induced by cerebral hypoperfusion in MMD/MMS patients may be the glymphatic system. The glymphatic system is a macroscopic waste clearance system that utilizes a unique system of perivascular tunnels to promote efficient elimination of soluble proteins and metabolites from the central nervous system (21). Through this system, cerebrospinal fluid (CSF) from the subarachnoid space moves rapidly into the perivascular spaces surrounding penetrating cerebral arteries, exchanging with brain interstitial fluid (ISF) and facilitating the clearance of interstitial solutes (22). Previous reports have suggested that this exchange may be driven by arterial pulsation (21). Therefore, arterial stenosis in MMD/MMS patients might result in reduced perfusion of large vessels, which would affect the function of the glymphatic system, block the exchange and circulation of cerebrospinal fluid and brain interstitial fluid, and accelerate the accumulation of metabolic substances in PVS, further leading to dilation of the PVS. This hypothesis captures the potential correlation between large artery stenosis and small cerebral vessel disease but needs to be validated in further longitudinal studies.

The methodological strengths of the present study include the large sample size of cross-sectional data and the quantitative and validated measurement of brain volumes. Our study also has potential limitations. First, due to the cross-sectional design of this neuroimaging study, the exact pathogenesis of the dPVS observed in MMD/MMS was not clarified. Second, we have not further assessed data on cerebral blood perfusion from unilateral cerebral hemispheres or discussed its relationship with dPVS, which means that we cannot offer direct support for our hypothesis regarding the relationship between CSO-dPVS and vascular stenosis. Third, we enrolled both MMD and MMS patients in the present study. The latter term refers to a characteristic cerebrovascular condition similar to MMD with the presence of other definite etiologies or co-existing diseases, including atherosclerosis. Our findings may suggest a correlation between PVS and intracranial artery stenosis to some extent. However, since the specific etiological and risk factors of MMS have not been classified in this study, large-sample studies are needed to further verify the correlation. Finally, HR VWI but not digital subtraction angiography (DSA) was used for all patients in our cohort. Compared to DSA, HR VWI has been repeatedly demonstrated to be a reliable, non-invasive method to evaluate intracranial arterial stenosis and collateral circulation. However, previous findings have illustrated that HR VWI shows a strong correlation with DSA (23, 24).

## 5. Conclusion

In conclusion, CSO-dPVS burden was found to be independently associated with degree of MCA stenosis in MMD/MMS, but not with ipsilateral hemisphere volume. These results indicate an important direct association between intracranial large and small vessels, which might contribute to a better understanding of the mechanisms responsible for dPVS in MMD/MMS.

## Data availability statement

The raw data supporting the conclusions of this article will be made available by the authors, without undue reservation.

## Ethics statement

The studies involving human participants were reviewed and approved by Ethics Committee of Peking Union Medical College Hospital. Written informed consent to participate in this study was provided by the participants themselves or, if not applicable, by their legal guardian/next of kin.

## Author contributions

GH contributed to the analysis, interpretation of the data, the study design, and the writing of the manuscript. XF and YH contributed to the analysis and interpretation of the data. LZ, YZ, and FF contributed to the collection and interpretation of data. MY and JN contributed to the collection, analysis, interpretation of the data, the study design, and the revision of the manuscript. All authors have read and approved the final manuscript.
